# Classification and Authentication of Lonicerae Japonicae Flos and Lonicerae Flos by Using ^1^H-NMR Spectroscopy and Chemical Pattern Recognition Analysis

**DOI:** 10.3390/molecules28196860

**Published:** 2023-09-28

**Authors:** Kaishuang Liu, Yibao Jin, Lifei Gu, Meifang Li, Ping Wang, Guo Yin, Shuhong Wang, Tiejie Wang, Lijun Wang, Bing Wang

**Affiliations:** NMPA Key Laboratory for Quality Research and Evaluation of Traditional Chinese Medicine, Shenzhen Institute for Drug Control, Shenzhen 518057, China; kerain1109@163.com (K.L.); jinyibao2006@126.com (Y.J.); liphiegu@gmail.com (L.G.); szlimeifang@126.com (M.L.); wangping662@sina.com (P.W.); ayinguoa@126.com (G.Y.); wangshuhongszyj@163.com (S.W.); szyjwtj@163.com (T.W.)

**Keywords:** ^1^H-NMR spectroscopy, Lonicerae japonicae flos, Lonicerae flos, chemical pattern recognition, quality evaluation

## Abstract

Lonicerae japonicae flos and Lonicerae flos are increasingly widely used in food and traditional medicine products around the world. Due to their high demand and similar appearance, they are often used in a confused or adulterated way; therefore, a rapid and comprehensive analytical method is highly required. In this case, the comparative analysis of a total of 100 samples with different species, growth modes, and processing methods was carried out by nuclear magnetic resonance (^1^H-NMR) spectroscopy and chemical pattern recognition analysis. The obtained ^1^H-NMR spectrums were employed by principal component analysis (PCA), partial least-squares discriminant analysis (PLS-DA), orthogonal partial least-squares discriminant analysis (OPLS-DA), and linear discriminant analysis (LDA). Specifically, after the dimensionality reduction of data, linear discriminant analysis (LDA) exhibited good classification abilities for the species, growth modes, and processing methods. It is worth noting that the sample prediction accuracy from the testing set and the cross-validation predictions of the LDA models were higher than 95.65% and 98.1%, respectively. In addition, the results showed that macranthoidin A, macranthoidin B, and dipsacoside B could be considered as the main differential components of Lonicerae japonicae flos and Lonicerae Flos, while secoxyloganin, secologanoside, and sweroside could be responsible for distinguishing cultivated and wild Lonicerae japonicae Flos. Accordingly, ^1^H-NMR spectroscopy combined with chemical pattern recognition gives a comprehensive overview and provides new insight into the quality control and evaluation of Lonicerae japonicae flos.

## 1. Introduction

Lonicerae japonicae flos (LJF), the dried bud or flower with initial blooming of *Lonicera japonica* Thunb., is not only one of the most widely used traditional Chinese medicines (TCMs) but has also been widely used as a food or dietary supplement by people for thousands of years in China [1,2]. In clinical settings, LJF is commonly used in the treatment of carbuncles, furuncles, febrile fever, and so on for its clearing heat and detoxification functions [3]. What is more, it is also an important constituent for various traditional Chinese medicine preparations [4]. There are more than 500 frequently used preparations containing LJF in the cure of diseases [5,6]. Many of them, such as the Lianhuaqingwen capsule/granula that contain LJF, have shown satisfactory efficacy in preventing severe acute respiratory symptoms in 2003, influenza A/H1N1 in 2009, and especially the novel coronavirus (COVID-19) that broke out in 2019 [7,8,9].

Drug and food adulteration refers to the fraudulent and purposeful substitution or addition of some other substances to a product to enhance its apparent value or reduce its cost. With the increasing prevalence of adulteration, modern detection and quality control methods have gradually become more and more important [10,11]. Due to the excellent pharmaceutical activities of LJF, the consumption of LJF is increasing largely. The phytochemical constituents of LJF are species-dependent and can significantly vary according to the geographical location, wild-harvested or cultivated, processing method, and cultivation method. However, Lonicerae flos (LF, *Lonicera macrantha* (D.Don) Spreng.) is often found to be substituted or adulterated in LJF for historical or commercial reasons. Since 2005, the *Chinese Pharmacopoeia* listed LJF and LF as two independent items based on their different plant morphology, chemical composition, and medicinal properties [12]. It has been reported that LF is rich in saponins which can cause an immediate hypersensitivity reaction in drug injection [13]. Thus, for the safety of clinical medication, especially in drug injection, LJF is forbidden to be substituted or adulterated by LF [14,15]. However, the two kinds of traditional Chinese medicine have morphological similarity; they are not able to be easily and clearly identified. Even worse, people often use LF instead of LJF due to the high yields and low price of LF. In this situation, it is essential to solve the problem of the mixed use of LJF to ensure its efficiency and safety in clinical settings.

Over the past few decades, there have been many advances in the quality control of LJF and LF. For example, Shi et al. established a quality control method for LJF and LF using the chemical fingerprints of seven major compounds and antibacterial effects based on ultra-high performance liquid chromatography (UHPLC) and microcalorimetry [1]. Zhao et al. developed rapid screening and quantitative analysis methods of adulterant LF in LJF by Fourier-transform near-infrared spectroscopy (FT-NIRs) and chemometrics [16]. Cai et al. provided a quality evaluation approach of LJF and LF based on 50 multiple bioactive constituent contents determined by UFLC-QTRAP-MS/MS and multivariate statistical analysis [17]. What is more, Gu et al. [18] and Xie et al. [19] provided tools to discriminate LJF according to species, growth modes, processing methods, and geographical origin with UHPLC and chemical pattern recognition. Most of the methods mentioned above used the contents of several active substances as the evaluation standard, which is limited to reflecting the overall quality of herbals. However, traditional Chinese medicine should be treated as a whole rather than relying on one or more of the main labeled compounds. Thus, it is necessary to focus on a new comprehensive, effective, and environmentally friendly method for the quality control of TCM. Nuclear magnetic resonance (NMR) technology, as one of the modern, accurate diagnostic methods, relies on the response of all proton-bearing compounds in the sample to achieve qualitative and quantitative analysis, which offers some advantages over other methods such as fast analysis, simple in sample preparation, it being highly robust and highly reproducible, having stronger specificity, not requiring specific reference substances, and not relying on chromatographic separation [20]. NMR can collect all the chemical information of TCM, which is consistent with the holistic idea of TCM and can be a more comprehensive and effective tool for quality control [21]. In the past few years, NMR has been widely used in the quality control [22] and authenticity identification of many kinds of TCM [23,24]. However, to the best of our knowledge, there are no reports on the identification of LJF and LF by combining ^1^H-NMR spectroscopy with chemical pattern recognition.

In this study, the application of the ^1^H-NMR spectroscopy method coupled with chemical pattern recognition analysis for the profiling of LJF samples with different growth modes, processing methods, and LF is reported. All of the information data obtained from ^1^H-NMR spectra were used for the establishment of chemometrics models. Partial least-squares discriminant analysis (PLS-DA) and orthogonal partial least-squares discriminant analysis (OPLS-DA) were applied to screen out the specific variations. The S-lines contributed to finding out the main differential components of different species and growth modes. Linear discriminant analysis (LDA) exhibited good classification abilities based on the specific variation in the classification of LJF with different species, growth modes, and processing methods. The established method is fast, environmentally friendly, and reproducible and can be a comprehensive quality evaluation method of LJF and LF without specific references.

## 2. Results and Discussion

### 2.1. Optimization of Sample Preparation

**Figure 1:**
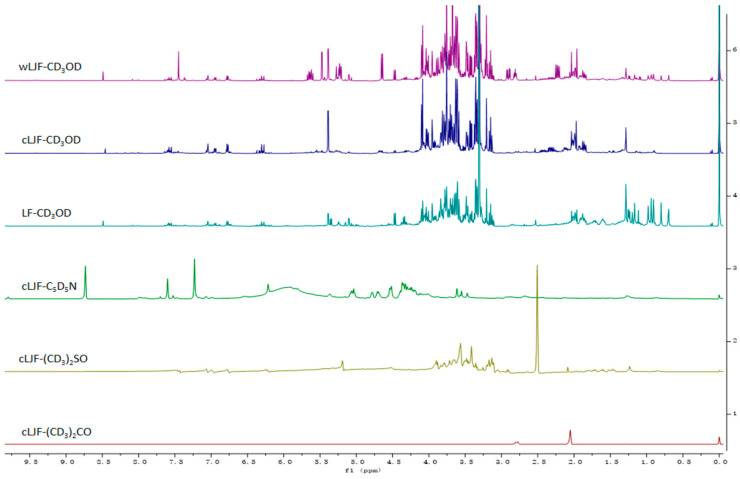
Typical ^1^H-NMR spectrums of wild Lonicerae japonicae flos (wLJF), Lonicerae flos (LF), and cultivated Lonicerae japonicae flos (cLJF) with different NMR solvents.

**Figure 2:**
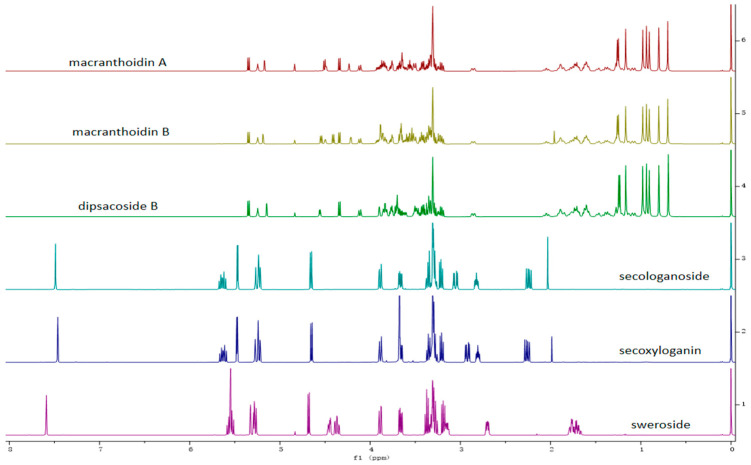
The reference substance ^1^H-NMR spectrums of macranthoidin A, macranthoidin B, dipsacoside B, secoxyloganin, secologanoside, and sweroside.

To obtain more useful chemical information, the conditions for the cultivated Lonicerae japonicae flos (cLJF) samples were optimized by comparing NMR solvents (CD_3_OD, (CD_3_)_2_CO, (CD_3_)_2_SO, and C_5_D_5_N). The results suggested that the CD_3_OD as the NMR solvent obtained the most chemical information compared with the other solvents. Therefore, CD_3_OD was considered as the optimum condition and applied equally to wLJF and LF (Figure 1). Six standard references, macranthoidin A, macranthoidin B, dipsacoside B, secologanoside, secoxyloganin and sweroside were also determined using the same experimental conditions as the samples. Their ^1^H-NMR spectrums were shown in Figure 2.

### 2.2. Identification and Analysis of Different Species and Growth Modes

#### 2.2.1. Principal Component Analysis (PCA)

**Figure 3:**
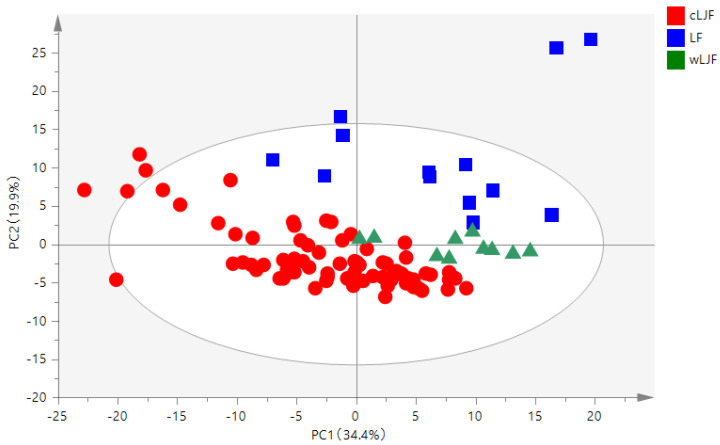
Score plot of the PCA based on the species and growth modes.

To reduce the dimensionality of the multivariate data while preserving most of the variance within them, the PCA method was used, which is an unsupervised clustering method requiring no knowledge of the data set. In this study, A PCA analysis was performed based on the obtained data matrix with dimensions 100 (samples) × 200 (integrated values) using SIMCA-P 14.0 software. The analysis showed that the normalized integrate value matrix was transformed into principal components (PCs) for analysis, and 13 PCs were obtained, which extracted and explained 95.7% (R^2^X = 0.957) of the variance, and the predictive ability (Q^2^) of the model was 86.9% (Q^2^ = 0.869). The PCA score plot revealed that all the samples were divided into three separate groups, where LJF and LF could be distinguished, indicating that species is the main factor affecting *Lonicera* quality (Figure 3). However, wLJF is closer to LF, which revealed that growth mode may affect *Lonicera* quality. The PCA model showed that several wLJF were mixed with cLJF and LF samples; therefore, the supervised pattern recognition method was needed to find out the specific variation accurately.

#### 2.2.2. Extraction of the Specific Variation

**Figure 4:**
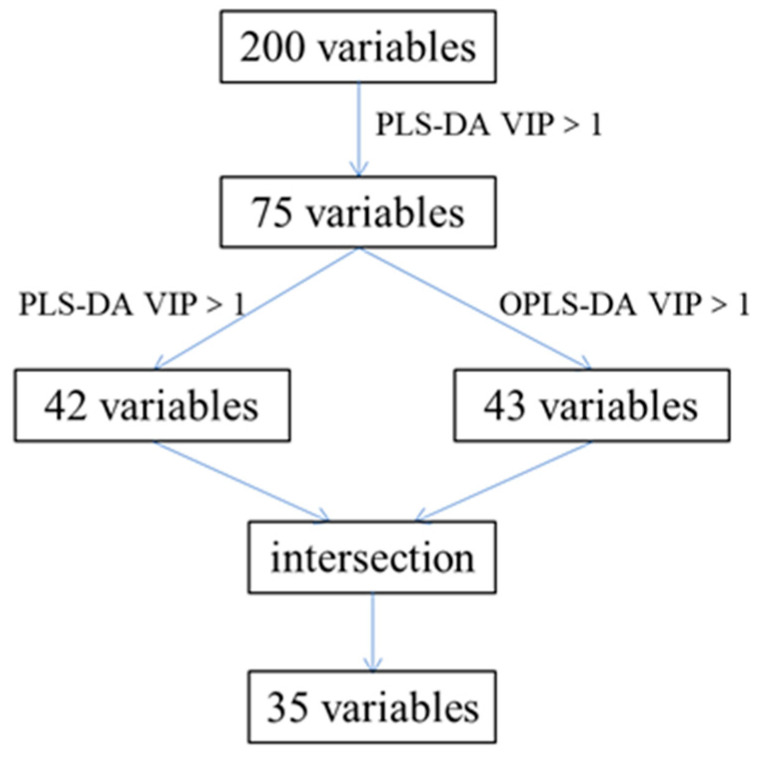
Flowchart for extraction of the specific variation.

When supervised pattern recognition research is carried out, samples are generally divided into a training set and a testing set. The training set was used to establish models and the testing set was used to verify the recognition accuracy and the predictive ability of the models [25]. In this study, 100 batches of samples were randomly divided, of which 47 batches of cLJF, 6 batches of wLJF, and 8 batches of LF were used as the training set, and the remaining 39 batches were used as the testing set. In order to accurately discriminate LJF and LF, PLS-DA and OPLS-DA were combined for the first time for the dimensionality reduction of the data. Figure 4 displays the details of the dimensionality reduction process.

PLS-DA was performed in SIMCA-P 14.0 software based on the data matrix with dimensions 61 (samples) × 200 (integrated values) of 61 batches of the training set. Variable importance in project (VIP), a commonly used variable importance in project, was selected to evaluate the contribution of the variables. The larger the VIP value of the variable, the greater its contribution to classification. Variables with VIP values greater than 1.0 can be distinguished as feature markers [26,27]. With VIP value > 1 as the screening criteria, 75 variables were selected in the PLS-DA model. After that, the data matrix of 61 (samples) × 75 (integrated values) was used to perform PLS-DA and OPLS-DA models, and 42 and 43 variables were obtained, respectively. At the intersection, 35 variables were selected as the characteristic variables, indicating their contribution to the accuracy of discrimination.

#### 2.2.3. Identification of the Characteristic Variables

**Figure 5:**
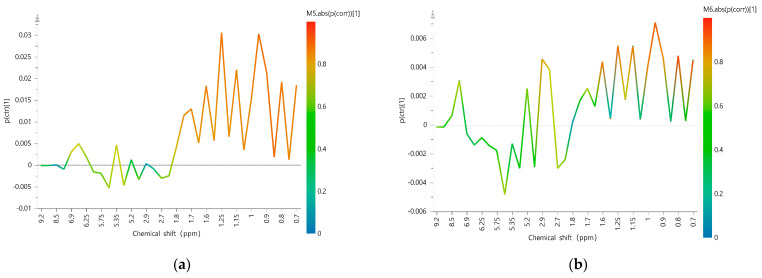
S-line obtained based on the OPLS-DA model between LJF and LF (**a**) and between cLJF and wLJF (**b**).

In order to identify the different characteristic variables between the groups, the S-lines analysis of the OPLS-DA models based on the selected 35 characteristic variables were obtained between LJF and LF on the one hand (Figure 5a) and between cLJF and wLJF on the other hand (Figure 5b). The results in Figure 5a indicate that the main difference between LJF and LF was found in the region between 0.65 and 1.80 ppm, which corresponds to the signals of macranthoidin A, macranthoidin B, and dipsacoside B. And they have positive intensities in LF compared to LJF (Figure 1 and Figure 2). Interestingly, when comparing wLJF with cLJF in a separate model, 0.65 and 1.80 ppm were also the main different regions as shown in the S-line (Figure 5b). However, in the current *Chinese Pharmacopoeia* [28] and most of the research [29], macranthoidin B and dipsacoside B are considered as characteristic components to evaluate the chemical quality of LF, whereas they are in trace amounts in LJF. Thus, our results indicate that when macranthoidin B and dipsacoside B are used as quality markers for LF, reasonable limits need to be set since they can also be detected in most wLJF. Besides, in order to avoid immediate hypersensitivity reactions, cLJF would be more suitable than wLJF when they are used as the raw material for drug injection production.

Previous research has proven that compared with cLJF, wLJF contains more secoxyloganin and secologanoside [19]. This can also be seen from the ^1^H-NMR spectra in our study. In the growth modes, other different characteristic variables are also found in the regions between 2.65 and 2.95 ppm, 4.60 and 4.70 ppm, and 5.20 and 5.30 ppm (Figure 5b) which represent the part signals of secoxyloganin and secologanoside (Figure 2). And the region of 5.50 and 5.55 ppm corresponds to the part signal of sweroside.

#### 2.2.4. Linear Discriminant Analysis (LDA)

The linear discriminant analysis system is robust in terms of classification, and it can also be used for dimension reduction or data visualization as well. It is a supervised machine learning method that computes decision boundaries in order to enhance separation between multiple classes, unlike PCA, which tries to maximize variance. A maximum distance between projected means and a minimal projected variance is used to distinguish different classes [25].

In this study, stepwise LDA was performed in SPSS 26.0 software based on the data matrix with dimensions 100 (samples) × 35 (integrated values), which used 61 batches of the training set and 39 batches of the testing set. Consequently, in order to generate discriminant functions, the LDA model finally selected seven characteristic variables, which denoted integrated values of variables V186, V184, V147, V145, V66, V31, and V17. Among the seven characteristic variables, V186, V184, V147, and V145 were in the regions of 0.65–1.80 ppm and 2.65–2.95 ppm. This further indicated that macranthoidin A, macranthoidin B, dipsacoside B, secoxyloganin, and secologanoside were the quality markers of the species and growth modes. The three discriminant functions of identification were as follows:

A = 245.678V17 − 321.509V31 + 71.423V66 + 19.730V145 + 176.163V147 + 471.194V184 − 954.988V186 − 10.103(1)

B = 311.308V17 + 1139.664V31 + 472.028V66 − 350.766V145 − 1371.149V147 + 8174.827V184 − 6550.292V186 − 92.660(2)

**Figure 6:**
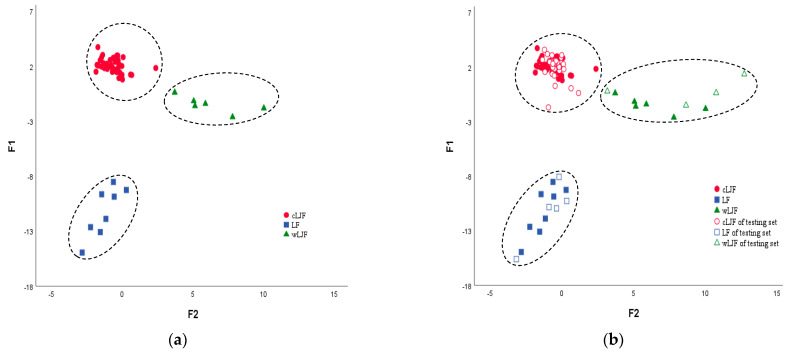
The classification models for samples based on the species and growth modes: (**a**) LDA score plot of the training set samples and (**b**) LDA score plot of the training set and testing set samples.

C = −4229.002V17 + 1114.710V31 + 35.912V66 + 434.342V145 − 452.648V147 + 1229.008V184 − 953.914V186 − 29.427(3)

where A is the classification function of cLJF, B is the classification function of LF, and C is the classification function of wLJF. All the training sets were divided into three separate regions (Figure 6a), indicating significant differences between the samples with different species and growth modes. The leave-one-out cross-validation method was used as a powerful parameter to predict the accuracy of the model; the LDA model correctly classified 100.0% of the samples. To validate the classification prediction performance of the established model, three discriminant functions were used to verify 39 batches of testing set samples. The results of the discriminant function values (Supplementary Tables S1 and S2) and the score plot (Figure 6b) of the samples showed that all samples were accurately divided into their categories. This indicated that the established LDA model in our study is reliable in distinguishing cLJF, wLJF, and LF samples.

#### 2.2.5. Verification of the Distinguishing Ability of the Characteristic Variables

**Figure 7:**
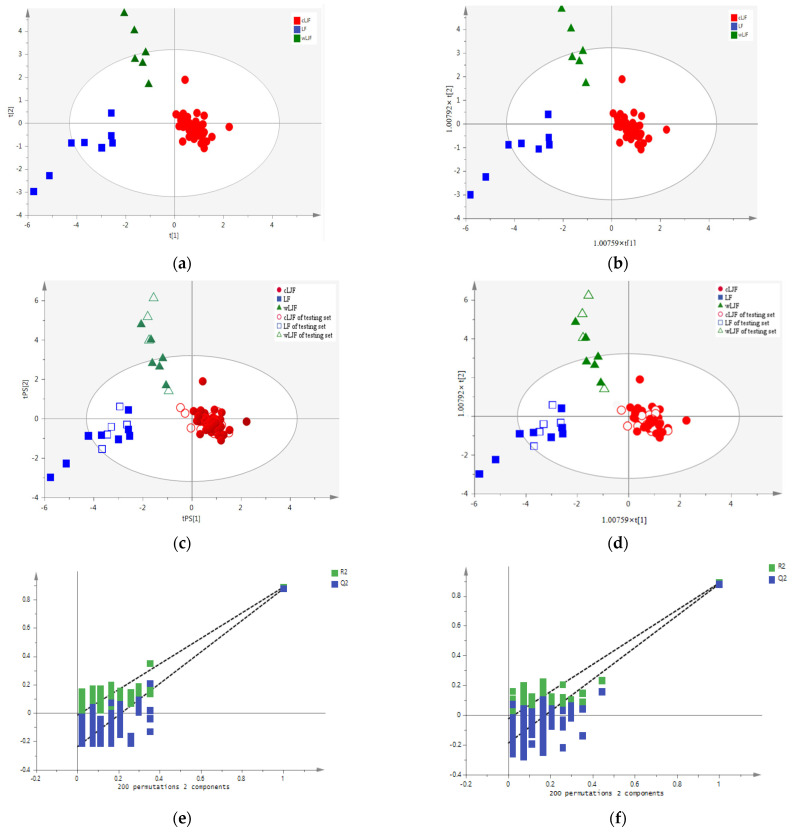
The classification models for the samples based on the species and growth modes. (**a**) PLS-DA score plot of the training set samples. (**b**) OPLS-DA score plot of the training set samples. (**c**) PLS-DA score plot of the training set and testing set samples. (**d**) OPLS-DA score plot of the training set and testing set samples. (**e**) Permutation test result of PLS-DA. (**f**) Permutation test result of OPLS-DA. Dotted line in (**e**,**f**) represent the regression line of R^2^ and Q^2^ in the permutation test.

To verify whether the seven variables can discriminate LJF and LF, PLS-DA and OPLS-DA models were performed in SIMCA-P 14.0 software. In the PLS-DA and OPLS-DA models, the data matrix with dimensions 100 (samples) × 7 (integrated values) was used. A clearer separation was achieved between the cLJF, wLJF, and LF samples, which can be seen in the score plots (Figure 7a,b). The validation results in the PLS-DA and OPLS-DA models show that all the testing set samples were correctly classified into the corresponding categories (Figure 7c,d). The models showed high values of R^2^X, R^2^Y, and Q^2^ (Table 1), in which R^2^X and R^2^Y (close to 1) indicated the good fitness of the model, whereas the high Q^2^ value (>0.5) showed the good predictivity of the model.

The PLS-DA and OPLS-DA models were validated using a permutation test to confirm the validity of the developed models. The 200 permutation tests were performed, and the vertical intercept values of R^2^ and Q^2^ of PLS-DA and OPLS-DA were (−0.0153, −0.238) and (−0.0247, −0.188), respectively, indicating that the models were not over-fitting (Figure 7e,f). In general, the accuracies of the LDA, PLS-DA, and OPLS-DA were all 100%, indicating their ability to distinguish the different species and growth modes of the samples based on the ^1^H-NMR spectra.

### 2.3. Identification and Analysis of Two Processing Methods

The processing method of TCM is also an important factor influencing its quality and yield [15]. Many studies have suggested that the plucking time and processing methods, influenced by nature, determine the quality and pharmacological effects of cLJF [30]. Therefore, it was necessary to discuss the influence of the processing methods on cLJF.

**Figure 8:**
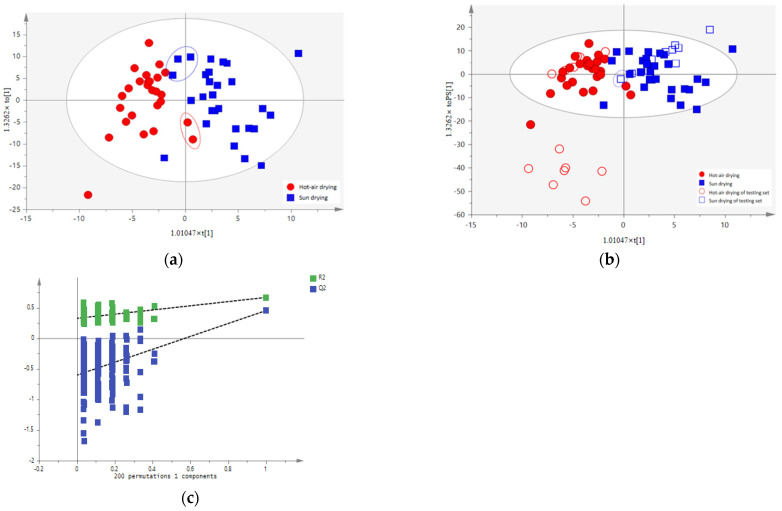
The classification models for the cLJF samples based on the two processing methods: (**a**) OPLS-DA score plot of the training set samples; (**b**) OPLS-DA score plot of the training set and testing set samples; and (**c**) permutation test result of the OPLS-DA. Dotted line in (**c**) represents the regression line of R^2^ and Q^2^ in the permutation test.

In order to investigate the effects of different processing methods on cLJF, OPLS-DA and LDA models were used to classify the cLJF samples. Firstly, the cLJF samples were divided into a training set, which contained 26 batches of hot air drying and 28 batches of sun drying, and the remaining 23 batches were divided into a testing set. Secondly, the OPLS-DA model was built. In the training step of OPLS-DA, two hot air drying samples were misclassified as sun-drying and three sun-drying samples were misclassified as hot-air drying (Figure 8a). In the testing step of OPLS-DA, one sun drying sample was misclassified as hot air drying (Figure 8b). With the R^2^X and Q^2^ of the OPLS-DA model, it indicated the good fitness and poor predictive ability of the model (Table 2). Finally, the 200 permutation tests were performed, and the vertical intercept values of R^2^ and Q^2^ of OPLS-DA were (0.331 and −0.612), respectively, indicating that the model was not over-fitting (Figure 8c).

**Figure 9:**
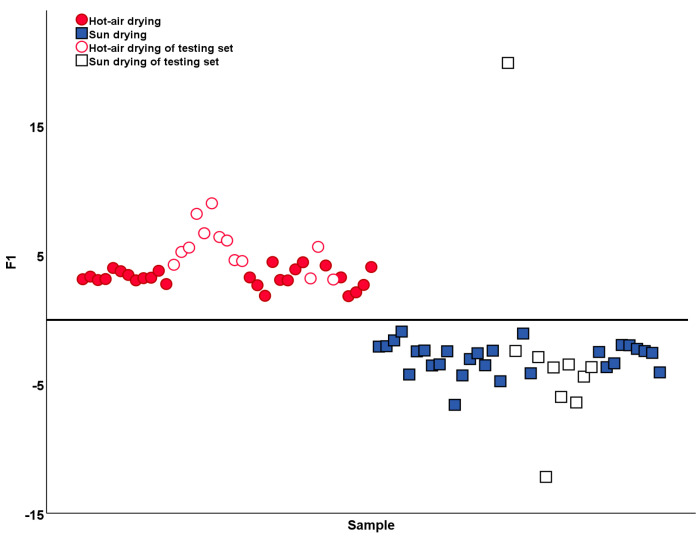
LDA score plot of the training set and testing set samples of cLJF based on the two processing methods.

Moreover, based on the variables, with the VIP value >1 in the OPLS-DA model, we attempted to establish a stepwise LDA model to further discuss the influence of the processing methods (Figure 9). The results of the LDA analysis of two processing methods showed that due to the misclassification of J47, the sample prediction accuracy from the training set was 100%, while the sample prediction accuracy from the testing set was 95.65%. The value of cross-validation accuracy was 98.1%. As a result, compared to OPLS-DA, the LDA model could be a more appropriate method to classify the processing methods of cLJF.

## 3. Materials and Methods

### 3.1. Materials and Reagents

A total of 100 batches of LJF and LF samples were collected from all over China, including 77 batches of cultivated Lonicerae japonicae flos (cLJF) with different processing methods and different origins, 10 batches of wild Lonicerae japonicae flos (wLJF), and 13 batches of Lonicerae flos (LF). The detailed sample information is shown in Table 3. All the samples were authenticated by Chief Pharmacist Ji Zhang, who is the former Director of the Traditional Chinese Medicine Herbarium at the China National Institute for Food and Drug Control. Voucher samples were preserved and stored in the cold sample room of Shenzhen Institute for Drug Control.

Deuterated methanol (CD_3_OD, purity ≥ 99.8%) containing 0.05% (*v*/*v*) TMS was purchased from Merck. Secologanoside (purity 99.28%) analytical standard was acquired from Sichuan Weikeqi Biological Technology Co., Ltd. Secoxyloganin (Chengdu, China) (purity 98.93%) analytical standard was acquired from Shanghai Hongyong Biological Technology Co., Ltd. Macranthoidin A (Shanghai, China) (purity 100.0%) was acquired from Standard Technology Co., Ltd. (Shanghai, China). Macranthoidin B (purity 96.1%), Dipsacoside B (purity 96.8%) and Sweroside (purity 98.3%) were purchased from the National Institute for Food and Drug Control (Beijing, China). All of the other chemicals used in this study were of analytical grade.

### 3.2. Sample Preparation

The aqueous extract of the samples was based on a previously reported method [18]. Briefly, each sample was accurately weighed at 6.00 g, soaked with 120 mL of water for 1 h, and extracted by reflux twice for 1 h each time. Then, the extract solution, filter, and concentrate were combined. After freeze-drying, the dried extracts were obtained. After drying for more than 4 h in a vacuum dryer with phosphorus pentoxide, 15 mg of dried extracts was accurately weighed and transferred into a 2.5 mL centrifuge tube. Then, 600 μL of methanol-d6 NMR solvent was added followed by ultrasonication for 1 min and centrifugation for 10 min at 13,500 rpm. The supernatant was taken and transferred to a 5 mm NMR tube for NMR measurement.

### 3.3. ^1^H-NMR Spectra Measurement

The ^1^H-NMR spectra were analyzed by a Bruker AV Ⅲ HD-500 NMR spectrometer with the experimental parameters based on the previous report with slight modification [31]. The instrument was operated at a proton NMR frequency of 500.13 MHz and acquired under automation at a constant temperature of 300 K. A presaturation sequence (ZGPR) was applied to suppress the residual solvent signal, of which the transmitter frequency offset was set for 4.842 ppm. For each sample, the ^1^H-NMR spectrum consisted of 128 scans with a spectral width of 8012.820 Hz and an acquisition time of 2.0447 s. The ^1^H-NMR spectra of 100 batches of samples and the six standard references were determined using the above experimental conditions, and the spectra were automatically Fourier transformed (Figure 1 and Figure 2).

### 3.4. Data Processing of the NMR Spectra

Automatic phase and baseline corrections were applied during spectra processing. The calibration of the data was performed by shifting the TMS signal to 0.0 ppm using MestReNova 9.0 software (version 14.2.0, Mestrelab Research, Santiago, Spain). The region of δ −0.025–9.975 in the nuclear magnetic resonance spectrum was integrated at the section of 0.05 ppm, thus producing 200 discrete bucketed regions. In order to eliminate the dimensional influence of each variable, all of the integrated values were normalized in relation to the peak of TMS signal intensity and scaled to 1.0. Then, a data set consisting of a 100 × 200 matrix was obtained for further chemometric analyses, in which rows represented samples and columns represented integrated values determined by NMR.

### 3.5. Multivariate Statistical Analysis

Chemical pattern recognition analysis can be described as the use of mathematical and statistical techniques to analyze several types of chemical data [32,33]. It includes unsupervised pattern recognition and supervised pattern recognition [34]. The most widely used method for unsupervised pattern recognition is principal component analysis (PCA), which only extracts important information from the data and is used to provide an intrinsic overview of the data set and reveal possible groups and outliers [35]. The commonly applied supervised pattern recognition methods are partial least-squares–discriminant analysis (PLS–DA), orthogonal partial least-squares discriminant analysis (OPLS-DA), and linear discriminant analysis (LDA). In supervised pattern recognition methods, the samples of prior known information are usually divided into a training set and a testing set.

The ^1^H-NMR spectrums were processed by MesrReNova 14.2.0 software (Mestrelab Research, Santigo, Spain). The normalized data matrix obtained from each sample was analyzed by SIMCA-P 14.0 software (Umetrics AB, Umea, Sweden) and SPSS 26.0 software (IBM, Chicago, IL, USA) for chemical pattern recognition. Simca-p14.0 software was used for the PCA, PLS-DA, and OPLS-DA models and SPSS 26.0 software was used for the LDA models.

## 4. Conclusions

In this study, the ^1^H-NMR spectrums of LJF and LF were obtained by ^1^H-NMR spectroscopy, and the possibility of discriminating the species and processing methods of LJF was investigated systematically with PCA, PLS-DA, OPLS-DA, and LDA models. The LDA results were highly satisfactory, with good cross-validated predictions (higher than 98.1%) and low classification errors (below 4.35%). This strongly demonstrated that the constructed method can serve as a powerful tool for distinguishing LJF and LF and classification of LJF from different processing modes without a reference substance and accurate determination of the content. The S-line further indicated that the 0.65–1.80 ppm and 2.65–2.95 ppm represented the ^1^H-NMR spectra profiles of macranthoidin A, macranthoidin B, dipsacoside B, secoxyloganin, and secologanoside, which can serve as a significant characteristic for distinguishing LJF and LF and the classification of LJF from different processing modes. Therefore, the ^1^H-NMR spectra profiles coupled with chemical pattern recognition provide a new tool in the comprehensive quality control of LJF and offer a favorable strategy to solve the problem of mixed use of LF.

## Figures and Tables

**Table 1:** Summary of classification results from the PLS-DA and OPLS-DA models of species and growth modes.

Model	Categories	Number of Samples		Number of Correct Classification (%)		R^2^X (cum)	R^2^Y (cum)	Q^2^ (cum)
		Training Set	Testing Set	Training Set	Testing Set			
PLS-DA	cLJF	47	30	61 (100%)	39 (100%)	0.647	0.839	0.821
	wLJF	6	4					
	LF	8	5					
OPLS-DA	cLJF	47	30	61 (100%)	39 (100%)	0.647	0.839	0.817
	wLJF	6	4					
	LF	8	5					

**Table 2:** Summary of classification results from the OPLS-DA model of the two processing methods.

Model	Categories	Number of Samples		Number of Correct Classification (%)		R^2^X (cum)	R^2^Y (cum)	Q^2^ (cum)
		Training Set	Testing Set	Training Set	Testing Set			
OPLS-DA	Hot air drying	26	13	54 (90.74%)	23 (95.65%)	0.793	0.671	0.456
	Sun drying	28	10					

**Table 3:** Detailed information of 100 batches of samples.

Sample No.	Species	Growth Modes	Processing Methods	Origins
J1–J3/J5/J9/J23–J29	Lonicerae japonicae flos	Cultivated	Hot-air drying	Henan
J4/J6–J8/J10–J15/J17–J18/J43–J53/J55–J58	Lonicerae japonicae flos	Cultivated	Sun drying	Shandong
J16/J34–J41	Lonicerae japonicae flos	Cultivated	Hot-air drying	Shandong
J19–J22/J30–J33	Lonicerae japonicae flos	Cultivated	Hot-air drying	Hebei
J42/J54/J59–J63	Lonicerae japonicae flos	Cultivated	Sun drying	Henan
J64–J68	Lonicerae japonicae flos	Cultivated	Sun drying	Hebei
J69–J71	Lonicerae japonicae flos	Cultivated	Hot-air drying	Anhui
J72–J73	Lonicerae japonicae flos	Cultivated	Hot-air drying	Hubei
J74	Lonicerae japonicae flos	Cultivated	Hot-air drying	Sichuan
J75	Lonicerae japonicae flos	Cultivated	Hot-air drying	Guizhou
J76	Lonicerae japonicae flos	Cultivated	Hot-air drying	Hunan
J77	Lonicerae japonicae flos	Cultivated	Hot-air drying	Gansu
J78	Lonicerae japonicae flos	Cultivated	Hot-air drying	Ningxia Hui Autonomous Region
Y1–Y10	Lonicerae japonicae flos	Wild	Sun drying	Hubei
S1–S2	Lonicerae Flos	Wild	Sun drying	Jiangxi
S3	Lonicerae Flos	Wild	Sun drying	Guizhou
S4–S7	Lonicerae Flos	Wild	Sun drying	Hunan
S8–S12	Lonicerae Flos	Wild	Sun drying	/
S13	Lonicerae Flos	Wild	Sun drying	Sichuan

## Data Availability

The data are available within this article.

## Supplementary Materials

The following supporting information can be downloaded at: https://www.mdpi.com/article/10.3390/molecules28196860/s1. Table S1: The discriminant function values of the training set samples. Table S2. The discriminant function values of the testing set samples.

## Sample Availability

Samples of the Lonicerae japonicae flos are available from the authors.

## References

[1] Shi Z., Liu Z., Ming C., Wu Q., Su H., Ma X., Zang Y., Wang J., Zhao Y., Xiao X. (2016). Spectrum-Effect Relationships Between Chemical Fingerprints and Antibacterial Effects of Lonicerae Japonicae Flos and Lonicerae Flos Base on UPLC and Microcalorimetry. Front. Pharmacol..

[2] Zhang F., Shi P., Liu H., Zhang Y., Yu X., Li J., Pu G. (2019). A Simple, Rapid, and Practical Method for Distinguishing Lonicerae Japonicae Flos from Lonicerae Flos. Molecules.

[3] Zheng S., Liu S., Hou A., Wang S., Na Y., Hu J., Jiang H., Yang L. (2022). Systematic review of Lonicerae Japonicae Flos: A significant food and traditional Chinese medicine. Front. Pharmacol..

[4] Cheng B.C.-Y., Ma X.-Q., Kwan H.-Y., Tse K.-W., Cao H.-H. (2014). A herbal formula consisting of Rosae Multiflorae Fructus and Lonicerae Japonicae Flos inhibits inflammatory mediators in LPS-stimulated RAW 264.7 macrophages. J. Ethnopharmacol..

[5] Miao H., Zhang Y., Huang Z., Lu B., Ji L. (2019). Lonicera japonica Attenuates Carbon Tetrachloride-Induced Liver Fibrosis in Mice: Molecular Mechanisms of Action. Am. J. Chin. Med..

[6] Shang X., Pan H., Li M., Miao X., Ding H. (2011). Lonicera japonica Thunb.: Ethnopharmacology, phytochemistry and pharmacology of an important traditional Chinese medicine. J. Ethnopharmacol..

[7] Liang Y.C., Long W.W., Qing Z.J., Rui B.Q., Yuan L.J., Ikhlas K., Rudolf B., An G.D. (2022). Traditional Chinese Medicines Against COVID19: A Global Overview. World Chin. Med..

[8] Hui Z., Sha Z., Li C., Qiang S., Maolun L., Han Y., Shan R., Tianqi M., Xianli M., Haibo X. (2021). Updated pharmacological effects of Lonicerae japonicae flos, with a focus on its potential efficacy on coronavirus disease-2019 (COVID-19). Curr. Opin. Pharmacol..

[9] Hui L., Ming Y., Ling T.Q., Yang H.X., Willcox M.L., Ping L.J. (2021). Characteristics of registered clinical trials on traditional. Chinese medicine for coronavirus disease 2019 (COVID-19): A scoping review. Eur. J. Integr. Med..

[10] Uncu O., Ozen B., Tokatli F. (2020). Authentication of Turkish olive oils by using detailed pigment profile and spectroscopic techniques. J. Sci. Food Agric..

[11] Tarighat M.A., Abdi G., Tarighat F.A., Bayatiyani K.S. (2023). Authentication and identification of Lamiaceae family with cyclic voltammetry fingerprint-PCA-LDA and determination of the used phenolic contents for classification using chromatographic analyses. Talanta.

[12] Chinese Pharmacopoeia Commission (2005). Pharmacopoeia of the People’s Republic of China.

[13] Yuan G., Rui H., Yixin H., Qiaoling F., Runlan C., Yun Q. (2018). Shuang-Huang-Lian injection induces an immediate hypersensitivity reaction via C5a but not IgE. Sci. Rep..

[14] Yan Z. (2014). Study on Quality Control of Lonicera Japonica Flos and Lonicera Flos.

[15] Xingyue Y., Yali L., Aijuan H., Yang Y., Xin T., Liyun H. (2017). Systematic review for geo-authentic Lonicerae Japonicae Flos. Med. Front..

[16] Jing Z., Pengdi C., Huan L., Chunhua W., Tongchuan S. (2020). Rapid screening and quantitative analysis of adulterant Lonicerae Flos in Lonicerae Japonicae Flos by Fourier-transform near infrared spectroscopy. Infrared Phys. Technol..

[17] Zhichen C., Chengcheng W., Cuihua C., Lisi Z., Chuan C., Jiali C., Mengxia T., Xunhong L. (2021). Quality evaluation of Lonicerae Japonicae Flos and Lonicerae Flos based on simultaneous determination of multiple bioactive constituents combined with multivariate statistical analysis. Phytochem. Anal..

[18] Lifei G., Xueqing X., Bing W., Yibao J., Lijun W., Jue W., Guo Y., Kaishun B., Tiejie W. (2022). Discrimination of Lonicerae Japonicae Flos according to species, growth mode, processing method, and geographical origin with ultra-high performance liquid chromatography analysis and chemical pattern recognition. J. Pharm. Biomed. Anal..

[19] Xueqing X., Lifei G., Wanyi X., Guo Y., Jue W., Yibao J., Lijun W., Bing W., Tiejie W. (2022). Integrating Anti-Influenza Virus Activity and Chemical Pattern Recognition to Explore the Quality Evaluation Method of Lonicerae Japonicae Flos. Molecules.

[20] Riswanto F.D.O., Windarsih A., Lukitaningsih E., Rafi M., Fadzilah N.A., Rohman A. (2022). Metabolite Fingerprinting Based on ^1^H-NMR Spectroscopy and Liquid Chromatography for the Authentication of Herbal Products. Molecules.

[21] Galvan D., Aguiar L.M.d., Bona E., Marini F., Killner M.H.M. (2023). Successful combination of benchtop nuclear magnetic resonance spectroscopy and chemometric tools: A review. Anal. Chim. Acta.

[22] Tao L., Xuan H., Qiaoqi L. (2017). Metabolomic Differentiation of Rhodiola Crenulata from Different Geographical Origins of Sichuan Province and Tibet, China. Pak. J. Bot..

[23] Marchev A.S., Koycheva I.K., Aneva I.Y., Georgiev M.I. (2020). Authenticity and quality evaluation of different Rhodiola species and commercial products based on NMR-spectroscopy and HPLC. Phytochem. Anal..

[24] Xuanhao L., Xiaobo W., Daoxin H., Shangyu Z., Jinsong S., Gang F., Yi Z. (2019). Metabolic Discrimination of Different Rhodiola Species Using ^1^H-NMR and GEP Combinational Chemometrics. Chem. Pharm. Bull..

[25] Oliveri P., Malegori C., Mustorgi E., Casale M. (2020). Qualitative pattern recognition in chemistry: Theoretical background and practical guidelines. Microchem. J..

[26] Xiaohua Z., Renjun L., Jingjing Z., Xiangdong Q., Kailong Y., Yaqian Z., Leyuan P., Jinfang N. (2023). Authentication of the production season of Xinyang Maojian green tea using two-dimensional fingerprints coupled with chemometric multivariate calibration and pattern recognition analysis. LWT.

[27] Hua D., Wenli C., Yutian L., Fengchao L., Huimin L., Wei D., Guihua J. (2021). Discrimination of authenticity of Fritillariae Cirrhosae Bulbus based on terahertz spectroscopy and chemometric analysis. Microchem. J..

[28] Chinese Pharmacopoeia Commission (2020). Pharmacopoeia of the People’s Republic of China.

[29] Li Y., Li W., Fu C., Song Y., Fu Q. (2020). Lonicerae japonicae flos and Lonicerae flos: A systematic review of ethnopharmacology, phytochemistry and pharmacology. Phytochem. Rev..

[30] Zhang Y.S.J., Jin Y., Bi Y.A., Wu H.X., Hao Q.X., Guo L.P., Xiao W. (2014). Standard operating procedure for raw materials of reduning injection—Lonicerae Japonicae Flos. Chin. Tradit. Herb. Drugs.

[31] Lu F.L., Wang L., Yan X.J., Chen Y.Y., Li D.P. (2019). ^1^H-NMR fingerprint identification for Lonicerae Japonicae Flos and Lonicerae Flos. Chin. Tradit. Pat. Med..

[32] Gao W., Yang H., Qi L.W., Liu E.H., Ren M.T., Yan Y.T., Chen J., Li P. (2012). Unbiased metabolite profiling by liquid chromatography-quadrupole time-of-flight mass spectrometry and multivariate data analysis for herbal authentication: Classification of seven Lonicera species flower buds. J. Chromatogr. A.

[33] Rohman A., Putr A.R. (2019). The Chemometrics Techniques in Combination with Instrumental Analytical Methods Applied in Halal Authentication Analysis. Indones. J. Chem..

[34] Wu X.D., Chen H.G., Zhou X., Huang Y., Hu E.M., Jiang Z.M., Zhao C., Gong X.-J., Deng Q.-F. (2015). Studies on Chromatographic Fingerprint and Fingerprinting Profile-Efficacy Relationship of Saxifraga stolonifera Meerb. Molecules.

[35] Cao X., Sun L., Li D., You G., Wang M., Ren X. (2018). Quality Evaluation of Phellodendri Chinensis Cortex by Fingerprint–Chemical Pattern Recognition. Molecules.

