# Supplementary material for: Classification and Authentication of Lonicerae Japonicae Flos and Lonicerae Flos by Using 1H-NMR Spectroscopy and Chemical Pattern Recognition Analysis
Source: Molecules. 2023 Sep 28;28(19):6860. doi: 10.3390/molecules28196860 (PMC10574709; doi:10.3390/molecules28196860)
Supplement: Supplementary file 1 [file molecules-28-06860-s001.zip › molecules-2615944-supplementary.pdf]

Classification and Authentication of Lonicerae Japonicae Flos and Lonicerae Flos by Using ^{1}H-NMR Spectroscopy and Chemical Pattern Recognition Analysis

Kaishuang Liu, Yibao Jin, Lifei Gu, Meifang Li, Ping Wang, Guo Yin, Shuhong Wang, Tiejie Wang, Lijun Wang * and Bing Wang *

NMPA Key Laboratory for Quality Research and Evaluation of Traditional Chinese Medicine, Shenzhen Institute for Drug Control, Shenzhen 518057, China
* Correspondence: wanglijun8706@hotmail.com (L.W.); wangbingszyj@163.com (B.W.)

Supplementary Tables

Table S1. The discriminant function values of the training set samples

No.	V17	V31	V66	V145	V147	V184	V186	A(cLJF)	B(LF)	C(wLJF)	Result
**J1**	0	-0.001	0.051	0.042	0.041	0.005	-0.003	7.13	-80.15	-20.02	A
**J2**	0	0.001	0.044	0.029	0.029	0.003	-0.004	3.63	-69.96	-19.76	A
**J3**	0	0.005	0.04	0.026	0.028	0.003	-0.002	-0.08	-77.97	-18.20	A
**J4**	-0.001	-0.001	0.063	0.045	0.051	0.005	-0.003	9.57	-89.56	-18.58	A
**J5**	0	0.003	0.052	0.037	0.04	0.005	-0.003	5.64	-71.99	-17.24	A
**J6**	-0.001	-0.001	0.054	0.026	0.023	0.001	-0.005	3.64	-68.35	-17.49	A
**J7**	0	0.006	0.064	0.039	0.051	0.004	-0.004	8.00	-80.32	-17.85	A
**J8**	-0.001	0	0.057	0.038	0.045	0.006	-0.003	8.09	-72.40	-16.78	A
**J9**	0.001	0.006	0.061	0.035	0.04	0.002	-0.005	6.02	-74.74	-20.45	A
**J10**	-0.001	0	0.05	0.037	0.047	0.004	-0.005	8.89	-81.34	-18.92	A
**J11**	0	0.012	0.042	0.056	0.05	0.004	-0.005	5.61	-81.91	-3.17	A
**J22**	0	0.005	0.045	0.035	0.036	0.004	-0.003	3.29	-75.01	-15.55	A
**J23**	0	-0.001	0.077	0.044	0.054	0.005	-0.005	13.23	-73.30	-22.19	A
**J24**	0.001	0.005	0.056	0.028	0.036	-0.001	-0.006	4.69	-88.27	-25.71	A
**J25**	0.002	0.003	0.056	0.044	0.047	0.005	-0.002	6.84	-88.09	-26.64	A
**J26**	0.001	0.004	0.057	0.037	0.041	0.004	-0.003	5.63	-77.73	-21.86	A
**J27**	0	-0.001	0.054	0.033	0.039	0.003	-0.003	5.88	-89.18	-25.37	A
**J28**	0.001	0.003	0.092	0.053	0.064	0.006	-0.006	16.63	-63.50	-19.86	A
**J29**	0	0.002	0.083	0.05	0.059	0.005	-0.006	14.65	-69.46	-17.34	A
**J30**	0	0.007	0.084	0.066	0.075	0.012	-0.002	15.72	-59.82	-7.23	A
**J31**	0.001	0.009	0.088	0.054	0.072	0.004	-0.008	16.81	-73.12	-17.05	A
**J32**	0.001	0.01	0.087	0.06	0.077	0.006	-0.005	15.49	-84.71	-16.03	A
**J33**	0.001	0.005	0.097	0.061	0.078	0.007	-0.005	18.48	-79.23	-20.04	A
**J34**	0.001	0.003	0.099	0.073	0.093	0.01	-0.004	22.60	-87.37	-21.04	A
**J35**	0.002	0.004	0.056	0.033	0.046	0.002	-0.005	7.57	-86.59	-30.68	A
**J36**	0.001	0.005	0.057	0.038	0.05	0.003	-0.004	7.40	-90.91	-24.66	A
**J37**	0.001	0.002	0.043	0.032	0.041	0.003	-0.003	4.70	-93.04	-27.99	A
**J38**	0	0.002	0.049	0.025	0.036	0	-0.005	4.36	-92.63	-26.11	A

**J39**	0.003	0.008	0.054	0.026	0.035	-0.004	-0.01	6.26	-81.43	-31.18	A
**J61**	0	0.001	0.053	0.037	0.047	0.003	-0.004	7.60	-93.20	-24.11	A
**J62**	0	0.001	0.047	0.031	0.039	0.003	-0.005	6.60	-76.41	-22.36	A
**J63**	0	-0.001	0.054	0.04	0.047	0.006	-0.003	8.84	-78.08	-22.27	A
**J64**	0.001	0.002	0.053	0.036	0.045	0.003	-0.005	8.11	-82.11	-25.80	A
**J65**	0	-0.001	0.043	0.033	0.042	0.005	-0.003	6.56	-82.14	-24.67	A
**J66**	0	0.001	0.045	0.034	0.042	0.003	-0.004	6.09	-89.07	-23.44	A
**J67**	0.001	0.002	0.051	0.032	0.042	0.003	-0.005	7.36	-77.53	-26.25	A
**J68**	0	-0.001	0.039	0.036	0.043	0.004	-0.003	6.04	-94.63	-25.19	A
**J69**	0.001	0	0.048	0.028	0.042	0.002	-0.004	6.28	-94.55	-32.51	A
**J70**	0	-0.001	0.038	0.028	0.035	0.003	-0.003	3.93	-89.50	-26.31	A
**J71**	0.001	-0.001	0.048	0.032	0.04	0.004	-0.003	6.32	-84.55	-29.48	A
**J72**	0.001	-0.003	0.054	0.035	0.041	0.004	-0.004	8.58	-79.87	-29.69	A
**J73**	0.001	0.003	0.043	0.033	0.039	0.003	-0.003	4.05	-89.51	-25.54	A
**J74**	0	-0.002	0.045	0.036	0.04	0.006	-0.002	6.25	-79.02	-23.23	A
**J75**	0	0.005	0.063	0.037	0.043	0.005	-0.003	6.32	-68.64	-15.98	A
**J76**	0	-0.001	0.049	0.034	0.04	0.003	-0.003	5.71	-93.27	-25.57	A
**J77**	0	-0.002	0.065	0.033	0.033	0.004	-0.002	5.44	-75.28	-23.10	A
**J78**	0.001	0.004	0.05	0.034	0.044	0.003	-0.003	5.13	-92.27	-26.00	A
**S2**	-0.001	0.008	0.039	0.027	0.012	0.024	0.007	-2.86	58.97	14.23	B
**S3**	0	0.002	0.13	0.026	0.012	0.045	0.027	-3.42	136.42	12.88	B
**S4**	0	0.002	0.101	0.044	0.013	0.029	0.007	6.61	115.25	18.62	B
**S7**	-0.002	0.006	0.161	0.025	0.002	0.022	0.006	4.46	118.58	22.77	B
**S9**	-0.001	0.001	0.107	0.029	0.003	0.018	0.003	3.69	71.89	10.26	B
**S11**	-0.001	0.003	0.09	0.026	0.004	0.018	0.005	0.04	52.72	8.21	B
**S12**	-0.002	0.001	0.101	0.029	0.004	0.026	0.008	2.19	100.02	18.88	B
**S13**	0	0.002	0.172	0.051	0.015	0.032	0.022	-0.74	69.84	12.68	B
**Y3**	-0.001	0.01	0.058	0.142	0.009	0.008	-0.001	-0.31	-44.40	56.42	C
**Y5**	-0.001	0.012	0.05	0.097	-0.001	0.004	-0.003	-4.15	-36.00	40.34	C
**Y7**	-0.001	0.01	0.051	0.08	0.011	0.004	-0.003	-1.66	-48.29	25.33	C
**Y8**	-0.002	0.009	0.04	0.056	0.011	0.004	-0.002	-3.79	-53.07	16.67	C
**Y9**	-0.001	0.007	0.045	0.059	0.025	0.008	0.001	-1.00	-59.88	7.41	C
**Y10**	-0.003	0.009	0.013	0.047	0.015	0.008	0	-5.47	-48.86	17.22	C

Table S2. The discriminant function values of the testing set samples

No.	V17	V31	V66	V145	V147	V184	V186	A(cLJF)	B(LF)	C(wLJF)	Result
**J12**	0	0.001	0.056	0.033	0.037	0.002	-0.006	7.42	-71.74	-20.53	A
**J13**	-0.001	-0.001	0.065	0.031	0.047	0.004	-0.003	8.26	-86.40	-24.01	A
**J14**	-0.002	0	0.045	0.037	0.027	0.006	-0.003	3.80	-53.34	-5.27	A
**J15**	-0.001	0	0.061	0.054	0.042	0.009	-0.002	8.62	-54.03	-5.60	A
**J16**	0	0.001	0.06	0.045	0.055	0.006	-0.003	10.13	-85.70	-21.27	A
**J17**	0	0.002	0.064	0.045	0.055	0.004	-0.006	12.02	-79.37	-19.61	A
**J18**	0	-0.002	0.068	0.052	0.061	0.006	-0.004	13.82	-89.47	-23.05	A

**J19**	0	0.001	0.049	0.034	0.032	0.004	-0.005	6.04	-58.74	-16.58	A
**J20**	0.001	-0.001	0.054	0.038	0.039	0.005	-0.003	7.16	-74.28	-24.97	A
**J21**	0.001	-0.001	0.049	0.039	0.041	0.004	-0.003	6.71	-87.91	-26.85	A
**J40**	0.001	-0.001	0.049	0.029	0.038	0.002	-0.004	5.99	-90.08	-31.34	A
**J41**	0.001	0.004	0.041	0.032	0.04	0.003	-0.002	2.79	-96.88	-26.34	A
**J42**	0	0.001	0.036	0.019	0.024	0	-0.006	2.48	-74.80	-23.91	A
**J43**	0.002	0.002	0.048	0.037	0.048	0.003	-0.003	6.64	-101.72	-33.04	A
**J44**	0	0.001	0.045	0.028	0.039	0.002	-0.004	4.97	-91.02	-25.91	A
**J45**	0.001	0.005	0.048	0.037	0.04	0.003	-0.003	4.02	-87.64	-21.85	A
**J46**	0.001	0.002	0.051	0.035	0.042	0.002	-0.005	6.95	-86.76	-26.18	A
**J47**	0	0.001	0.051	0.023	0.022	0.007	-0.002	2.76	-35.36	-15.94	A
**J48**	0.001	0.007	0.05	0.033	0.039	0.001	-0.006	5.19	-78.34	-20.42	A
**J50**	0.001	0.005	0.047	0.036	0.04	0.002	-0.005	5.37	-82.84	-21.64	A
**J51**	-0.001	-0.001	0.044	0.034	0.046	0.004	-0.003	6.64	-95.99	-23.01	A
**J52**	-0.001	-0.001	0.031	0.027	0.032	0.003	-0.004	3.59	-82.10	-20.45	A
**J53**	0	0	0.045	0.03	0.033	0.001	-0.006	5.72	-79.71	-22.77	A
**J54**	0	-0.001	0.045	0.035	0.041	0.005	-0.003	6.57	-80.53	-23.28	A
**J55**	0	-0.001	0.041	0.033	0.039	0.004	-0.003	5.42	-87.15	-24.61	A
**J56**	0	0	0.04	0.029	0.035	0.002	-0.004	4.25	-89.39	-24.96	A
**J57**	0	-0.003	0.043	0.036	0.043	0.004	-0.003	6.97	-95.02	-27.28	A
**J58**	0	-0.001	0.042	0.034	0.039	0.003	-0.004	5.99	-88.65	-24.42	A
**J59**	0	0.001	0.05	0.034	0.042	0.002	-0.005	6.93	-88.33	-23.53	A
**J60**	0	0	0.047	0.031	0.04	0.003	-0.004	6.15	-85.47	-24.88	A
**S1**	-0.001	0.011	0.044	0.026	0.014	0.026	0.008	-3.15	72.16	17.92	B
**S5**	0	0	0.118	0.053	0.015	0.036	0.009	10.38	159.22	26.70	B
**S6**	-0.001	0.004	0.114	0.031	0.004	0.023	0.008	1.02	84.66	15.64	B
**S8**	-0.002	-0.001	0.076	0.04	0.005	0.026	0.007	2.39	87.26	21.03	B
**S10**	-0.001	0.006	0.093	0.021	0.003	0.016	0.005	-1.93	44.33	7.49	B
**Y1**	-0.001	0.012	0.028	0.164	0.015	0.005	-0.002	-2.06	-90.20	61.68	C
**Y2**	-0.001	0.011	0.055	0.149	0.014	0.006	-0.002	0.19	-63.78	56.70	C
**Y4**	-0.001	0.007	0.052	0.124	0.006	0.007	-0.001	-1.13	-48.40	45.17	C
**Y6**	-0.001	0.007	0.041	0.047	0.023	0.007	0.001	-2.35	-62.99	1.73	C
